
JOURNAL INFORMATION
==============================
NLM Title Abbreviation: Tex Med J (Austin)
NLM Journal ID: 21130590R

Texas Medical Journal

ISSN: 0892-8495
Publisher: Biomedical Journal Digitization Project

ARTICLE INFORMATION
==============================
PMCID: PMC9625870
Article version: 1
Subjects: Books and Magazines

The Diseases of Society (the Vice and Crime Problem)

Print publication date: 1905 Jul
Volume: 21
Issue: 1
First page: 29
Last page: 30
License: This work is free of known copyright restrictions. For more information, please see PMC Back Issue Digitization.
License URL: https://creativecommons.org/publicdomain/mark/1.0/

==============================

Phillips' Emulsion 50% best NORWAY COD LIVER OIL minutely sub-divided, WITH WHEAT PHOSPHATES (Phillips')

Palatable, Permanent, Miscible in Water, Milk, Wine, etc.

Phillips' Milk of Magnesia Mg H2 O2 (FLUID.) "THE PERFECT ANTACID."

for correcting Hyperacid conditions—local or systemic.
Vehicle for Salicylates, Iodides, Balsams, etc.

Phillips' Phospho-Muriate of Quinine, COMP.

TONIC AND RECONSTRUCTIVE.

WHEAT PHOSPHATES, WITH MURIATE OF QUININE AND STRYCHNINE.

PHILLIPS' WHEAT PHOSPHATES (ACID).
PHILLIPS' SYRUP OF WHEAT PHOSPHATES.
PHILLIPS' DIGESTIBLE COCOA.

THE CHAS. H. PHILLIPS CHEMICAL CO., NEW YORK

years of clinical study. Throughout the work the value of the knowledge of topographic anatomy in bedside diagnosis is emphasized. The many colored lithographic plates and the numerous text-cuts, sixty of which are in colors, are of exceptional excellence. Indeed, both for accuratenesss of detail and artistic beauty, we have never seen their equal. The greater portion of the dissections from which these illustrations have been made are from the author's own preparations. Dr. George D. Stewart in editing the work has added many valuable notes.

---

THE DISEASES OF SOCIETY (The Vice and Crime Problem). By G. Frank Lydston, M. D., Professor of Genito-Urinary Surgery, State University of Illinois. Cloth. Pp. 624. Price, $3.00 net. Philadelphia: J. P. Lippincott Co. 1904.

The reading of this remarkable book has given me much satisfaction. I have thought many of the same thoughts, and I thoroughly agree with the talented author in most of his views. It is a strong, fearless and clear exposition of the defects in our social system—our so-called "civilization." It is virile and manly. But what are you going to do about it? Some of the evils may be remedied, but "reform" is out of the question. The "bad" principle in man is the survival of the brute instincts inherited from our pethecanthropoid ancestors. Evolution is not complete; these individuals, criminals, perverts, etc., are not always degenerate, they simply have not been evolved out of the semi-brute state. Dr. Lydston is a charming writer, and as clear as he is forceful. He dares to say what he thinks. He says it well, and for the most part he thinks correctly. The book is "alive," instinct with vigorous thought, "from kiver to kiver," as old Jucklins used to say, and if anyone who reads this wants to enjoy a "refreshing season" as the preachers say of "grace," let him get the book and read it. D.

---
